# The moderating effect of resilience on the relationship between academic stress and school adjustment in Korean students

**DOI:** 10.3389/fpsyg.2022.941129

**Published:** 2023-01-09

**Authors:** Sumi Choi, Inhwa Yoo, Dongil Kim, Sohyun An, Yoonhee Sung, Changhyun Kim

**Affiliations:** ^1^Department of Education, Konkuk University, Seoul, Republic of Korea; ^2^Department of Education, Seoul National University, Seoul, Republic of Korea; ^3^Department of Counseling Psychology, Gangseo University, Seoul, Republic of Korea

**Keywords:** academic stress, resilience, academic adjustment, general school adjustment, moderating effect

## Abstract

**Introduction:**

This study investigated the moderating effects of resilience on the relationship between academic stress and school adjustment among Korean adolescents. We examined the moderating effect for the total scores of school adjustment and the two subscales of school adjustment (academic adjustment and general school adjustment).

**Methods:**

Data were obtained in Pusan, Korea, using the convenience sampling method, and a total of 674 participants' responses were used for the final analysis. Hierarchical regression analysis and multiple regression analysis were performed to examine our research questions.

**Results:**

The results of the study showed that academic stress was a significant predictor of school adjustment among Korean adolescents. More specifically, academic stress was related to poor school adjustment. The study also found that resilience has a significant mediating effect on the relationship between academic stress and school adjustment.

**Discussion:**

Our findings suggest that resilience is likely to alleviate the harmful effect of academic stress on school adjustment. Finally, implications for school-based intervention were discussed for providing practical academic assistance.

## 1. Introduction

The impact of stress on the well-being of adolescents has been extensively documented. Although various levels and aspects of stress affect adolescents, the stress related to academics is one of the most significant stressors due to their developmental stage and accompanying expectations. In Asian societies, such as South Korea, China, and Japan, the level of academic stress is more severe (Ang and Huan, 2006). In South Korea, parents and teachers tend to put extremely high pressure on children regarding academic excellence in order to be competitive in future job markets. It is reported that Korean adolescents spend ~14–18 h per day studying at school and in private education (Lee and Larson, 2000). This disproportionately large amount of study hours could be a significant stressor in itself. According to the National Youth Policy Institute (2013), Korean adolescents reported academic stress as the biggest stressor in their lives. Choi and Lee (2010) conducted an extensive survey comparing various physical and psychological indicators of well-being among adolescents in Korea, the United States, Japan, and China. The results indicated that Korean adolescents reported the most frequent incidents of experiencing stress in a given year and that the main source of their stress was related to academics. Although learning is one of the most important developmental tasks of adolescents, in the academic context, learning can become a grueling process that could induce tremendous stress.

Such high academic stress has an array of effects on the well-being of adolescents. Research suggests that extreme academic stress among adolescents may lead to adverse psychological symptoms such as depression, anxiety, and suicidal ideation (e.g., Lee and Larson, 2000; Akgun and Ciarrochi, 2003). Suicide is one of the three major causes of death among adolescents and young adults worldwide (United Nations, 1996). The same is also true in Korea where academic stress is the most common cause of adolescent suicide (Kim and Chun, 2000; Park and Jung, 2010). Ang and Huan (2006) reported a significant mediating effect of depression between academic stress and suicidal ideation.

While the relationship between academic stress and negative mental health has been consistently reported, research on the impact of academic stress on school adjustment presents mixed results. Many studies have indicated links between high academic stress and various negative adjustment outcomes such as truancy (Kim, 1999), poor academic performance (Akgun and Ciarrochi, 2003), difficult relationships with peers and teachers (Lee, 2012), and poor academic achievement (Lee and Lee, 2010). Although excessive stress associated with learning impedes academic performance and even results in many students dropping out of school (Ahn and Yoo, 2014), Tung and Chahal (2005) found a weak direct association between stress and adjustment. Selye (1978) reported that academic stress does not necessarily lead to negative consequences, while some students suffering from academic stress may present the detrimental issues stated above, others maintain a state of stable equilibrium and exhibit sound school adjustment. Kang and Lee (2005) suggested that the effect of stress on the adjustment outcomes of students could be further explained by mediating and/or moderating factors, such as individual traits, personality, social support, and coping skills.

Resilience is a relatively versatile concept that has been examined in relation to overcoming various adversities in life. Historically, the concept originated from investigating individuals who were initially in the midst of adverse life challenges, such as severe mental health issues, and had shown better adjustment (Garmezy, 1970; Glick and Zigler, 1986). As the construct of resilience developed over the years, its definition has been approached in various ways, examining resilience as a trait, process, or outcome (Fletcher and Sarkar, 2013). Although a unified definition of resilience would yield tremendous advancement in the body of research on resilience, the variations in definition have not yet been resolved (Luthar et al., 2000).

One of the common approaches to defining resilience is viewing it as a cluster of personality traits. Connor and Davidson (2003) defined resilience as ‘the personal qualities that enable one to thrive in the face of adversity.' From this point of view, resilience has also been defined as the ability to adapt to situational demands in a flexible manner (Klohnen et al., 1996) and the cognitive capacity to deal with negative outcomes resulting from stress (Tugade et al., 2004). In general, highly resilient individuals tend to adapt well psychosocially (Park and Jung, 2010), and exhibit more positive emotions and higher self-confidence in the face of adversities than individuals with low resilience (Tugade et al., 2004). Compas et al. (1995) found that highly resilient students demonstrate high levels of academic achievement and easily obtain support from parents and teachers. Such individuals function well at school and tend to not show signs of delinquent behaviors and mental health problems (Robins et al., 1996).

In Korea, Kim et al. (2005) reported that highly resilient students experience less test-related stress than those with low resilience. In addition, students with high resilience experience fewer psychosomatic symptoms when test-related stress is high. Lee and Park (2002) found that highly resilient adolescents attend school and engage in social activities more actively than adolescents with low resilience. Therefore, resilience is a positive personality construct that may protect students from academic stress. The moderating effect of resilience on the link between academic stress and school adjustment among middle-school students was tested and confirmed in a Chinese sample (Han, 2012). Although resilience has mostly been examined in relation to stress in Korea (Jung et al., 2012), not many studies have assessed the role of resilience in relation to academic stress and school adjustment. To bridge this gap in existing literature, this study focuses on the moderating effect of resilience on the relationship between academic stress and school adjustment of Korean adolescents. To this effect, this study aims to investigate whether academic stress has a negative impact on school adjustment and whether the effect is moderated by resilience, based on the assumption that high levels of resilience would decrease the negative outcomes of academic stress on school adjustment. Therefore, this study examines the effect of the interaction between academic stress and resilience on school adjustment using a moderation model, which also allows testing the main effects of academic stress and resilience on school adjustment.

### 1.1. Purposes and research questions of the current study

Based on the discussion above, the present study investigated whether academic stress would have a negative relationship with school adjustment and whether the relationship would be moderated by resilience. We hypothesized high levels of resilience would decrease the negative outcomes of academic stress on school adjustment. In examining the moderating effect of resilience, we first tested the moderating effect for the total score of school adjustment (SA) and then examined the moderating effects of resilience for each of the two subscales of SA, which are academic adjustment(aa) and general school adjustment(gsa).

Our research hypotheses are as follows:

1. Resilience has a moderating effect on the relationship between academic stress and school adjustment(SA) in South Korean adolescents.
2. Resilience has a moderating effect on the relationship between academic stress and academic adjustment(aa), a subscale of school adjustment.
3. Resilience has a moderating effect on the relationship between academic stress and general school adjustment(gsa), a subscale of school adjustment.

## 2. Material and methods

### 2.1. Participants and procedure

#### 2.1.1. Participants

Data for this study were obtained anonymously toward the end of the academic year from middle and high schools in the Pusan province of South Korea using the convenience sampling method. Of 713 participants who initially completed the survey, 39 respondents were eliminated for the reason of incomplete responses (5.4%, *n* = 39), and a total of 674 responses were included in the analysis. Of the final 674 questionnaires, 336 students (49.9%) were girls and 338 students (50.1%) were boys, with a mean age of 16.78 years (SD = 1.61). A total of 301 students (44.7%) were enrolled in Korean middle schools (14.5%, 7th grade; 14.4%, 8th grade; 15.7%, 9th grade). In addition, a total of 373 students (55.3%) were enrolled in Korean high schools (18.1%, 10th grade; 23.6%, 11th grade; 13.6%, 12th grade).

#### 2.1.2. Participants and procedure

This study surveyed middle- and high-school students in Pusan, a metropolitan city and port in South Korea. A researcher contacted teachers or principals at the schools selected through convenient sampling and explained the purpose of the research and the survey procedure. For schools that consented to cooperate, instructions on how to administer the survey were provided by phone, and hard-copy surveys were sent to teachers *via* mail.

The instructed teachers distributed and administered the survey to students who agreed to participate in the survey after they were informed about the purpose of the study and asked for consent to collect data. Participants were requested to respond honestly in the process of conducting the survey in order to minimize the demand effect. The surveys completed by the participants were sent back to the researcher by mail.

### 2.2. Instruments

#### 2.2.1. Academic stress

Academic stress was measured using the Scale of Academic Stress (SAS), a self-report instrument, developed and validated by Park and Park (2012). Items of this scale were developed with some modifications of existing academic stress scales in consideration of the Korean education context. Participants were asked to rate the level of stress they experience in three different domains (academic performance, class, and studying) from three different sources of stress (parents, teachers, and oneself). A total of 45 items were used for the 3 domains × 3 sources of stress. Items were asked on a six-point Likert scale ranging from “Not at all” (1) to “Very much” (6). Example items for each domain are: “I am angry when the results of a test do not meet the expectations of my parent (or teacher, myself)” (academic performance domain); “I am afraid that my teacher would ask me a question that I cannot answer” (class domain); “I am stressed about the amount of studying that I have to do” (studies domain). The internal consistency reliability coefficient for this scale was 0.97 (specifically, 0.93 for stress related to academic performance, 0.93 for stress related to class, and 0.93 for stress related to studies).

#### 2.2.2. Resilience

Resilience was assessed using the Connor-Davidson Resilience Scale (CD-RISC) (Connor and Davidson, 2003). The CD-RISC is a self-report scale measuring the ability to cope with stress and adversity. This scale consists of 25 items and measures participants' level of resilience on a 5-point Likert scale, with higher scores reflecting high levels of resilience. Sample items include “I am able to handle unpleasant or painful feelings like sadness, fear and anger,” “I can deal with whatever comes,” and “When things look hopeless, I don't give up.” The scale is multidimensional and consists of five constructs: personal competence/tenacity, trust in one's instincts/tolerance for negative affect, positive acceptance of change/secure relationships, control, and spirituality. The internal consistency reliability coefficient for the scale was 0.92 in Connor and Davidson's study (2003) and 0.95 in the current study.

#### 2.2.3. School adjustment

The School Adjustment Scale was developed by Choi (1997) in consideration of the context of the Korean school system and was used to assess academic adjustment and levels of participation in various class activities. The scale consists of two subscales, academic adjustment (7 items) and general school adjustment (3 items). The participants were asked to rate how often they are engaged in the behavior each item describes using a five-point Likert Scale ranging from “Never” (1) to “Always” (5). Example items are as follows: “I attend classes diligently,” “I am trying to prepare and review thoroughly,” and “I actively participate in classroom activities such as group discussions.” The internal consistency reliability coefficient of the scale was 0.84 in Choi's (1997) study and 0.93 (0.91 for academic adjustment and 0.80 for general school adjustment) in the current study.

### 2.3. Ethical considerations

Most research institutions in South Korea, including the institutions to which the authors are affiliated, do not require IRB approval for social science studies yet. There are, however, common rules of research ethics in South Korea such as anonymous participants, providing participants with sufficient information including the purposes and the procedures of the research, and the voluntary nature of participation. These rules were duly followed.

### 2.4. Data analysis

Descriptive statistics and zero-order correlations were computed for all measures using the PASW 23.0 software. Next, hierarchical regression analysis was performed on hypothesis #1, with resilience specified as a moderator of the relationship between academic stress and school adjustment. The general procedure for fitting linear models was followed by using the PROCESS tool (Hayes, 2012). In particular, the centered predictor (academic stress) and centered moderator (resilience) were first specified, followed by creating the term of interaction, and finally, multiple regression analysis was implemented. Meanwhile, for examining hypotheses #2 and #3, with the moderating effects of resilience on the relationship between academic stress and two subscales of school adjustment (aa and gsa), multiple regression analysis was conducted using SAS 9.4 software.

## 3. Results

### 3.1. Descriptive and correlational analyses

Table 1 lists the means and standard deviations for all the variables measured. Following Fidell et al. (1996), the normality test resulted in the data meeting the criteria suitable for further analysis. In addition, to check for the multicollinearity issue, the tolerance values indicated that none of the independent variables indicated a value <0.10, which suggests no serious collinearity problem (Menard, 2002). Furthermore, no variable indicated a variance inflation factor (VIF) value >10 in this study, which indicates no evidence of multicollinearity between the variables included in the model (Myers, 1990).

**Table 1:** Descriptive statistics of variables.

	**Minimum**	**Maximum**	**Mean**	**Std. dev**.
Academic stress (AS)	1.00	5.00	3.50	1.003
Resilience (RE)	1.24	5.00	3.37	0.733
School adjustment (SA)	1.00	5.00	3.12	0.838
Academic adjustment (aa)	1.00	5.00	3.06	0.864
General school adjustment (gsa)	1.00	5.00	3.24	0.924

Std. dev., Standard deviation.

The zero-order correlations and *p*-values between the variables used in this study are provided in Table 2. Academic stress was found to be negatively related to resilience, and school adjustment and resilience were positively associated with school adjustment.

**Table 2:** Zero-order correlations of variables.

	**1**	**2**	**3**	**4**	**5**	**6**
1. Academic stress	—					
2. Resilience	−0.443^**^	—				
3. School adjustment	−0.387^**^	0.767^**^	—			
4. Academic adjustment	−0.384^**^	0.729^**^	0.978^**^	—		
5. General school adjustment	−0.332^**^	0.727^**^	0.889^**^	0.774^**^	—	

^**^, ^*^Represent the significance level of 5, 10% respectively (^**^p <0.05, ^*^p <0.01).

### 3.2. Moderating effect of resilience on the relationship between academic stress and school adjustment

Hierarchical regression was used to examine research hypotheses, with resilience specified as a moderator of the relationship between an independent variable (Academic Stress, AS) and dependent variables (School Adjustment, SA; academic adjustment, aa; and general school adjustment, gsa). AS, Resilience (RE), interaction terms of AS and RE, and control variables (gender and grade) were entered simultaneously for the regression analysis.

The results indicated that the grade of the student, which is a control variable, had a significant effect on school adjustment, academic adjustment, and general school adjustment. Therefore, the interaction effect of resilience, which is the control variable (grade and gender), was additionally analyzed; however, no significant impact was observed in the interaction effect.

Related to research hypothesis #1, AS had a significantly negative effect on SA and the interaction term (AS x RE) had a significant positive effect on SA (β = 0.06, *p* < 0.05) when controlling for grade and gender in Table 3. Particularly, a low level of RE (−0.73) had a significant slope between AS and SA (β = −0.10, *p* < 0.01), a mean level of RE (0.00) had a significant slope (β = −0.05, *p* < 0.05), and a high level of RE (0.73) had no significant slope (β = 0.01, *p* = 0.73). The resulting graphs from the moderating effects of RE are illustrated in **Figure 2**.

**Table 3:** Moderating effect of RE between AS and SA.

**DV**	**IV**	***b***	**se**	***t***	***P***
SA	Constant	1.65	0.39	4.291	0.000
	AS	−0.26	0.09	−2.969	0.003
	Resilience	0.60	0.10	5.939	0.000
	AS × resilience	0.06	0.02	2.558	0.011
	Grade	−0.20	0.04	−4.819	0.000
	Gender	−0.03	0.04	−0.736	0.462

AS, Academic stress; SA, School adjustment.

#### 3.2.1. Moderating effect of resilience on the relationship between academic stress and two subscales of school adjustment (aa and gsa)

**Figure 1:**
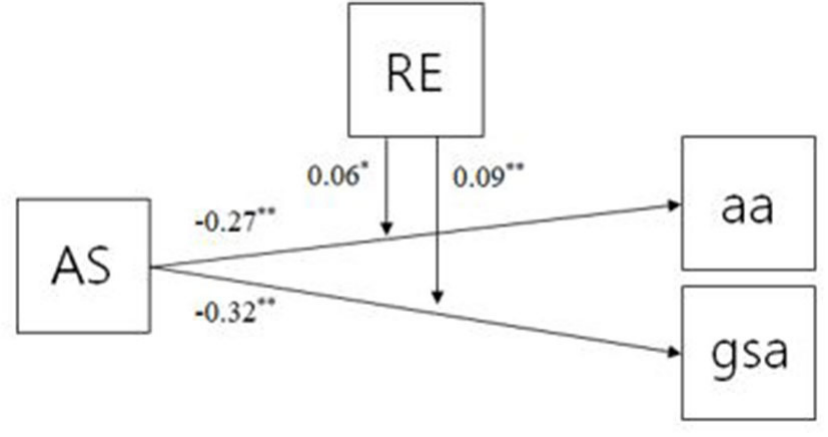
Moderation model of RE between AS and two subscales of SA (aa and gsa). ^**^, ^*^Represent the significance level of 5, 10% respectively (^**^p < 0.05, ^*^p < 0.01).

The research hypothesis # 2 and # 3 are shown in Figure 1, AS and RE were statistically significant effects on aa. However, two variables had different effects on aa, that is, AS negatively influenced aa (β = −0.27, *p* < 0.01) and RE had a positive effect on aa (β = 0.59, *p* < 0.0001). Meanwhile, AS and RE had a statistically significant effect on gsa. AS had a significant negative influence on gsa (β = −0.32, *p* < 0.01) and RE had a positive effect on it (β = 0.55, *p* < 0.0001). The interaction terms (AS x RE) had significantly positive effects on aa (β = 0.06, *p* < 0.05) and gsa (β = 0.09, *p* < 0.001).

**Figure 2:**
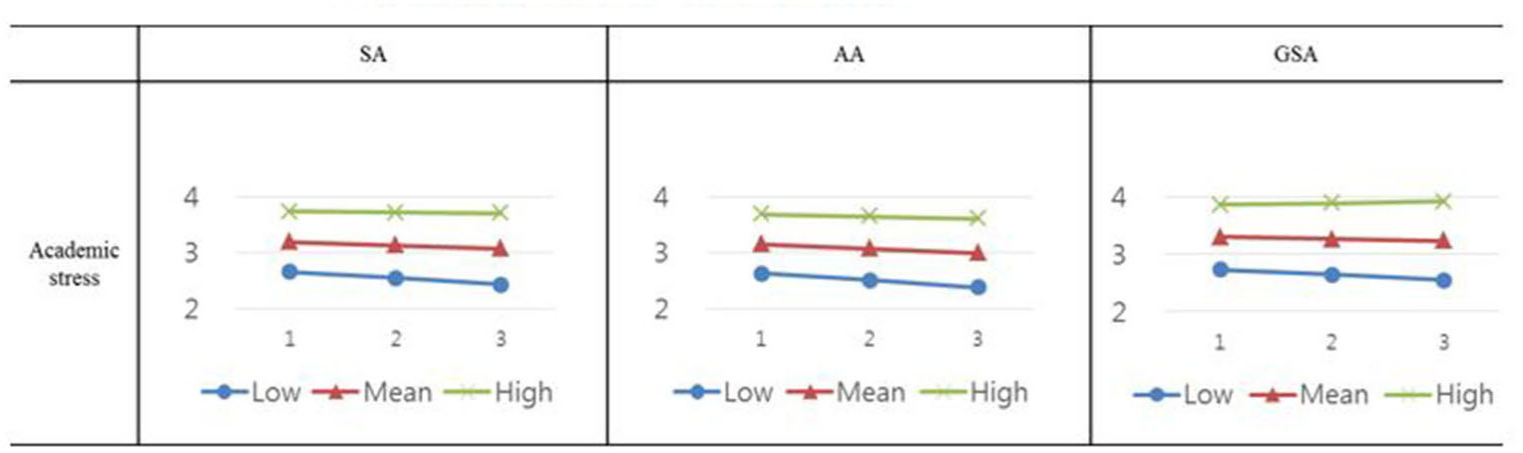
Regression plot displaying the main effects of resilience and academic stress (AS) × resilience interaction effects on school adjustment (SA, aa, and gsa).

These are shown in Figure 2. Particularly, a low level of RE (−0.73) had no significant slope between AS and gsa (β = −0.08, *p* < 0.05), a mean level of RE (0.00) had no statistically significant slope (β = −0.02, *p* = 0.42), and high resilience level of RE (0.73) had no significant slope (β = 0.04, *p* = 0.20) either.

## 4. Discussion

This study aimed to examine the effects of academic stress on school adjustment (i.e.SA, aa, and gsa) among Korean adolescents moderated by resilience. The negative impact of academic stress on school adjustment and the effect of the interaction between academic stress and resilience on school adjustment were investigated. The results showed a significant negative effect of academic stress on school adjustment among Korean adolescents, confirming our research hypothesis. This finding is compatible with previous research findings (Lee, 2004, 2012; Lee and Lee, 2010) indicating that academic stress is an important predictor of school adjustment among Korean adolescents.

The findings of this study further revealed that resilience moderated the relationship between academic stress and school adjustment. These findings suggest that the negative impact of academic stress on school adjustment was likely to be tempered by resilience and that students with high resilience are likely to have better school adjustment despite academic stress. This result is similar to the findings of a previous study by Han (2012) on Chinese students. Han (2012) found that resilience served as a buffer against academic stress among Chinese junior high-school students, resulting in improved school adjustment. In addition, previous research on resilience has shown that highly resilient individuals successfully adjust by responding to situational demands flexibly, with the ability to mitigate tension or exercise patience (Block and Kremen, 1996). These findings confirm that resilience protects individuals from external stimuli that may hamper adjustment.

The results of this study suggest two possible interventions—decreasing the negative effects of academic stress itself and resilience-based intervention. For instance, studies on social and emotional learning (SEL) suggest that interventions targeting SEL competencies such as self- and social awareness, responsible decision-making, and self- and relationship management may help reduce social and emotional strains from academic stress while cultivating academic achievement (Zins et al., 2004).

Another medium of promoting school adjustment would be enhancing the resilience of students. Resilience is not a static state of existence as disputed by Luthar et al. (2000); Waller (2001); Mahoney and Bergman (2002), and Ungar (2008). Newman (2005) asserted that various aspects of resilience i.e., behaviors, thoughts, and characteristics, can be cultivated and acquired. Psychoeducational programs and counseling interventions nurturing and utilizing various protective and promotive factors should be devised in supporting students at risk of academic stress and maladjustment. The focus on individual resilience would mitigate the maladjustment symptoms stemming from academic stress. Identifying individual traits and processes involved in resilience is needed to develop effective and strength-based interventions (Catalano et al., 2011).

Future research in this field is needed to enable generalizations regarding the protective role of resilience in the relationship between academic stress and school adjustment among Korean adolescents. Other variables, such as family support and level of mental health, need to be considered for a comprehensive evaluation of the role of resilience in the optimal development of adolescents. An adolescent student's academic success is the result of a combination of personality traits and environmental support (Masten, 2001). Since most adolescents are still under the care of family and educational institutions, attending to contextual factors that hinge on student engagement in learning and academic functioning would be important areas for school intervention (Hoagwood et al., 2007). These factors include a positive family–school relationship (Christenson and Havsy, 2004), proactive classroom management (Gettinger and Kohler, 2015), and constructive teacher–student relationships (Flay et al., 2001). Lee (2012) suggests that the effect of resilience on school adjustment among Korean high-school students is mediated by the family environment. Therefore, investigating the role of factors related to the family environment, such as parenting practices and socioeconomic conditions, would be significant in understanding the adjustment of adolescents.

Although the present study reveals important findings, it has some limitations. With respect to measuring school adjustment, this study used a self-report measure, a subjective assessment of adjustment. Although subjective assessment of adjustment is meaningful in that it reveals how an individual perceives and evaluates their own adjustment, integrating objective measures will provide a more comprehensive understanding of school adjustment. In addition, since convenience sampling was conducted in a specific region, there are limitations in generalizing the results of this study. Therefore, follow-up studies comprising diversified regions are recommended in the future.

## 5. Conclusion

Academic stress is an inevitable factor in the prediction of school maladjustment among adolescents. However, the impact of academic stress on school adjustment may vary depending on whether the negative effects of academic stress have been buffered. The results obtained in this study and existing literature concur that resilience is a valuable resource to mitigate academic stress among Korean middle- and high-school students and improve their school adjustment in the face of academic stress. In addition, the results suggest that resilience could serve as a coping resource for Korean adolescents, enabling them to develop optimally and adjust well in school. This is especially true for students experiencing excessive academic stress to the extent of considering or committing suicide. In order to help students to manage their academic stress, it may be advisable to first assess their resilience levels and provide professional help to boost their resilience. Psychoeducational programs and professional counseling interventions against academic stress should focus on utilizing individual resilience to alleviate the symptoms of maladjustment stemming from academic stress.

More studies in this field are needed to enable generalizations regarding the protective role of resilience in relation to academic stress and school adjustment among Korean adolescents. Nevertheless, the present study emphasizes the importance of resilience within the educational context. Furthermore, the study proposes the implementation of measures aimed at improving resilience among students who experience difficulties in school adjustment, as well as an evaluation of the effects of such measures. Hoagwood et al. (2007) suggest that psychological counseling and support services should categorically, fiscally, structurally, and scientifically operate in alliance with academic programs in schools. Academic stress can easily lead to mental health concerns among students, and therefore, schools must act diligently to offer interventions that target both stress and resilience, and, at the same time, provide practical academic assistance to students.

## Data availability statement

The raw data supporting the conclusions of this article will be made available by the authors, without undue reservation.

## Ethics statement

Ethical review and approval was not required for the study on human participants in accordance with the local legislation and institutional requirements. Written informed consent to participate in this study was provided by the participants' legal guardian/next of kin.

## Author contributions

SC was responsible for funding requisition and the study design. DK contributed to the review and editing of the manuscript. SA, SC, and DK was responsible for methodology. YS and CK contributed to data collection and project administration. IY and SA contributed to the formal analysis. IY and SC contributed in writing-review and editing of the manuscript. All authors read and approved the final manuscript.

## References

[B1] Ahn J. H. Yoo M. H. (2014). Comparison of academic stress, stress coping and academic burnout between elementary gifted students and general students and analysis of the relationships. J. Gifted/Talent. Educ. 24, 169–189. 10.9722/JGTE.2014.24.2.169

[B2] Akgun S. Ciarrochi J. (2003). Learned resourcefulness moderates the relationship between academic stress and academic performance. Educ. Psychol. 23, 287–294. 10.1080/0144341032000060129

[B3] Ang R. P. Huan V. S. (2006). Relationship between academic stress and suicidal ideation: testing for depression as a mediator using multiple regression. Child Psychiatry Hum. Dev. 37, 133–143. 10.1007/s10578-006-0023-8 16858641

[B4] Block J. Kremen A. M. (1996). IQ and ego-resiliency: conceptual and empirical connections and separateness. J. Pers. Soc. Psychol. 70, 349–361. 10.1037/0022-3514.70.2.349 8636887

[B5] Catalano D. Chan F. Wilson L. Chiu C. Y. Muller V. R. (2011). The buffering effect of resilience on depression among individuals with spinal cord injury: a structural equation model. Rehab. Psychol. 56, 200–211. 10.1037/a0024571 21843016

[B6] Choi D. S. (1997). Analysis of relationship between the vocational personalities and related behaviors of high school student: Academic achievement, adjustment to school, and occupational values (*Master's thesis*). Seoul: Seoul National University.

[B7] Choi I. J. Lee K. B. (2010). Korean Adolescents Index V: The International Comparative Studies of Adolescent Health among Korea the United States. Bethesda, MD: National Institutes of Health.

[B8] Christenson S. L. Havsy L. H. (2004). Family-School-Peer Relationships: Significance for Social, Emotional and Academic Learning. New York, NY: Teachers College Press.

[B9] Compas B. E. Hinden B. R. Gerhardt C. A. (1995). Adolescent development: pathways and processes of risk and resilience. *Annu. Rev*. Psychol. 46, 265–293. 10.1146/annurev.ps.46.020195.001405 7872731

[B10] Connor K. M. Davidson J. R. T. (2003). Development of a new resilience scale: the Connor-Davidson Resilience Scale (CD-RISC). *Depress*. Anxiety. 18, 76–82. 10.1002/da.10113 12964174

[B11] Fidell S. Silvati L. Howe R. Pearsons K. S. Tabachnick B. Knopf R. C.. (1996). Effects of aircraft overflights on wilderness recreationists. J. Acoust. Soc. Am. 100, 2909–2918. 10.1121/1.417102 8914306

[B12] Flay B. R. Allred C. G. Ordway N. (2001). Effects of the positive action program on achievement and discipline: two matched-control comparisons. Prev. Sci. 2, 71–89. 10.1023/a:1011591613728 11523754

[B13] Fletcher D. Sarkar M. (2013). Psychological resilience: a review and critique of definitions, concepts, and theory. Eur. Psychol. 18, 12–23. 10.1027/1016-9040/a000124

[B14] Garmezy N. (1970). Process and reactive schizophrenia: some conceptions and issues. Schizophr. Bull. 1, 30–74. 10.1093/schbul/1.2.30

[B15] Gettinger M. Kohler K. M. (2015). “Process-outcome approaches to classroom management and effective teaching,” in Handbook of Classroom Management. London,UK: Routledge.

[B16] Glick M. Zigler E. (1986). Premorbid social competence and psychiatric outcome in male and female nonschizophrenic patients. J. Consult. Clin. Psychol. 54, 402–403. 10.1037/0022-006X.54.3.402 3722573

[B17] Han Y. (2012). The moderating effects of ego-resilience on the relationship between academic stress and school adjustment of Chinese junior high school students (*Master's thesis*). Daegu: Kyungpook National University.

[B18] Hayes A. F. (2012). PROCESS: A versatile computational tool for observed variable mediation, moderation, and conditional process modeling (*Dissertation*). Ohio State: The Ohio State University.

[B19] Hoagwood K. E. Serene O. S. Kerker B. D. Kratochwill T. R. Crowe M. Saka N.. (2007). Empirically based school interventions targeted at academic and mental health functioning. J. Emot. Behav. Disord. 15, 66–92. 10.1177/10634266070150020301

[B20] Jung Y. E. Min J. A. Shin A. Y. (2012). The Korean version of the connor-davidson resilience scale: an extended validation: Validation of K-CD-RISC. Stress Health. 28, 319–326. 10.1002/smi.1436 23015460

[B21] Kang M. H. Lee S. Y. (2005). The mediating effects of hope and ego-resilience on the relationship between adolescents' academic stress and psychological well-being. Korean J. Youth Stud. 20, 265–293. 10.31782/IJCRR.2021.13528

[B22] Kim K. H. Kwon S. J. Shim M. Y. (2005). Test stress and the physical symptom of elementary school students: The moderating effect of ego-resiliency. Korean J. Health Psychol. 10, 113–126. Available online at: https://scholar.google.co.kr/scholar?hl=ko&as_sdt=0%2C5&q=Test+stress+and+the+physical+symptom+of+elementary+school+students%3A+The+moderating+effect+of+ego-resiliency&btnG=

[B23] Kim K. W. Chun M. H. (2000). Study on the teenage suicide. J. Korean Soc Child Welfare. 9, 127–152. Available online at: https://scholar.google.com/scholar_lookup?title=Study+on+the+teenage+suicide&publication_year=2000&journal=Journal+of+the+Korean+Society+of+Child+Welfare&pages=127-152

[B24] Kim K. Y. (1999). The relationship between stress in elementary school children and adaptation to family and school life (*Master's thesis*). Seoul: Sookmyung Women's University.

[B25] Klohnen E. C. Vandewater E. A. Young A. (1996). Negotiating the middle years: ego-resiliency and successful midlife adjustment in women. Psychol. Aging. 11, 431–442. 10.1037/0882-7974.11.3.431 8893312

[B26] Lee E. M. Park I. J. (2002). The relationships between parent-child bonding and children's ego-resiliency. Korean J. Family Welfare. 7, 3–24. Available online at: https://scholar.google.com/scholar?hl=ko&as_sdt=0%2C5&q=The+relationships+between+parentchild+bonding+and+children%E2%80%99+ego-resiliency&btnG=

[B27] Lee M. Larson R. (2000). The Korean examination hell: long hours of studying, distress, and depression. J. Youth Adolesc. 29, 249–271. 10.1023/A:1005160717081

[B28] Lee S. L. Lee S. J. (2010). A study on the effect of self-resilience on academic stress and school life adaptation. Educ. Res. 30, 85–113. Available online at: https://scholar.google.com/scholar?hl=ko&as_sdt=0%2C5&q=A+study+on+the+effect+of+self-resilience+on+academic+stress+and+school+life+adaptation&btnG=

[B29] Lee S. M. (2012). A study on the mediating effect of ego-resiliency, academic stress and mental health in the relationship between adolescent's family-environment and school adjustment. Korean J. Youth Stud. 19, 117–145. Available online at: https://scholar.google.com/scholar?hl=ko&as_sdt=0%2C5&q=A+study+on+the+mediating+effect+of+ego-resiliency%2C+academic+stress+and+mental+health+in+the+relationship+between+adolescent%E2%80%99s+family-environment+and+school+adjustment&btnG=

[B30] Lee Y. J. (2004). Effects of ego-resilience and parents conflict on school adjustment of elementary students. Korean J. Counsel. 5, 435–449. Available online at: https://scholar.google.com/scholar?hl=ko&as_sdt=0%2C5&q=Effects+of+ego-resilience+and+parents+conflict+on+school+adjustment+of+elementary+students&btnG=

[B31] Luthar S. S. Cicchetti D. Becker B. (2000). The construct of resilience: a critical evaluation and guidelines for future work. Child Dev. 71, 543–562. 10.1111/1467-8624.00164 10953923 PMC1885202

[B32] Mahoney J. L. Bergman L. R. (2002). Conceptual and methodological considerations in a developmental approach to the study of positive adaptation. J. Appl. Dev. Psychol. 23, 195–217. 10.1016/S0193-3973(02)00104-1

[B33] Masten A. S. (2001). Ordinary magic Resilience processes in development. Am. Psychol. 56, 200–211. 10.1037/0003-066X.56.3.227 11315249

[B34] Menard S. (2002). Applied Logistic Regression Analysis. Thousand Oaks, CA: Sage. 10.4135/9781412983433

[B35] Myers D. (1990). Housing Demography: Linking Demographic Structure and Housing Markets. University of Wisconsin Press.

[B36] National Youth Policy Institute (2013). A Study on Support Measures for Children and Adolescents'ental Health Promotion III. Available online at: https://www.nypi.re.kr/modedg/contentsView.do?ucont_id=CTX001006&menu_nix=6S33tBHc&srch_mu_lang=ENG (accessed May 5, 2022).

[B37] Newman R. (2005). APA's resilience initiative. Prof. Psychol. Res Pract. 36, 227–229. 10.1037/0735-7028.36.3.227

[B38] Park B. K. Park S. M. (2012). Development and validation of academic stress scales. Korean J. Educ. Psychol. Res. 26, 563–585. Available online at: https://www.kci.go.kr/kciportal/ci/sereArticleSearch/ciSereArtiView.kci?sereArticleSearchBean.artiId=ART001671433

[B39] Park S. Y. Jung Y. S. (2010). Moderating effects of ego-resilience and social support in relations among academic achievement pressure perceived academic stress and internalization problems in boys and girls. Korean J. Dev. Psychol. 23, 17–32. Available online at: https://scholar.google.co.kr/scholar?hl=ko&as_sdt=0%2C5&q=Moderating+effects+of+ego-resilience+and+social+support+in+relations+among+academic+achievement+pressure+perceived+academic+stress+and+internalization+problems+in+boys+and+girls&btnG=

[B40] Robins R. W. John O. P. Caspi A. Moffitt T. E. Stouthamer-Loeber M. (1996). Resilient, overcontrolled and undercontrolled boys: three replicable personality types. J. Pers. Soc. Psychol. 70, 157–171. 10.1037/0022-3514.70.1.157 8558407

[B41] Selye H. (1978). The Stress of Life. New York: McGraw-Hill.

[B42] Tugade M. M. Fredrickson B. L. Barrett L. F. (2004). Psychological resilience and positive emotional granularity: examining the benefits of positive emotions on coping and health. J. Pers. 72, 1161–1190. 10.1111/j.1467-6494.2004.00294.x 15509280 PMC1201429

[B43] Tung S. Chahal N. (2005). Relationship between stress and adjustment adolescent females: a casual study. J. Pers. Study Group Behav. 25, 19–31. Available online at: https://scholar.google.co.kr/scholar?hl=ko&as_sdt=0%2C5&q=Relationship+between+stress+and+adjustment+adolescent+females%3A+a+casual+study&btnG=

[B44] Ungar M. (2008). Resilience across cultures. *Br. J. Soc*. Work. 38, 218–235. 10.1093/bjsw/bcl34335076104

[B45] United Nations (1996). Prevention of Suicide: Guidelines for the Formulation and Implementation of National Strategies. New York: United Nations.

[B46] Waller M. A. (2001). Resilience in ecosystemic context: Evolution of the concept. Am. J. Orthopsychiatry. 71, 290–297. 10.1037/0002-9432.71.3.290 11495331

[B47] Zins J. E. Weissberg R. P. Wang M. C. Walberg H. J. (2004). Building Academic Success on Social and Emotional Learning: What Does the Research Say? (New York, NY: Teachers College Press), 59–75.

